# Effect of fluticasone-impregnated throat packs on postoperative sore throat (POST) and hoarseness of voice: A randomized clinical trial

**DOI:** 10.12688/f1000research.139742.1

**Published:** 2023-10-17

**Authors:** Arjun Talapatra, Shaji Mathew, Sushma Thimmaiah Kanakalakshmi, Rama Rani

**Affiliations:** 1 Department of Anaesthesiology, Kasturba Medical College, Manipal Academy of Higher Education, Manipal, Karnataka, 576104, India

**Keywords:** Endotracheal intubation, Fluticasone furoate, Hoarseness of voice, Nasosinus surgeries, Postoperative sore throat

## Abstract

**Background:** Post-operative sore throat (POST) is one of the most common complaints post-endotracheal intubation and can be decreased through various interventions. This study aimed to determine the effect of fluticasone-impregnated *versus* saline throat packs on the occurrence and severity of POST and voice hoarseness.

**Methods:** This prospective, randomized, double-blinded trial was conducted on patients undergoing nasosinus surgeries at Kasturba Medical College and Hospital. Patients were randomized to groups based on a computer-generated table of random numbers post-intubation after placing a definite length of oropharyngeal packs into group F (fluticasone) who received four puffs of fluticasone furoate-soaked throat packs and group C (control) wherein normal saline-soaked throat packs were used. Determining the incidence of POST and voice hoarseness was the primary outcome; severity of POST and voice hoarseness, patient satisfaction scores at 24 hours post-surgery and adverse events were secondary outcomes.

**Results:** Overall, 86 patients were randomized and 43 patients were included in each group. Incidence of POST (%) and voice hoarseness (%) were 55.8, 55.6, 55.8, 53.4 and 30.2, 28, 28, 28 in group C. Incidence of POST (%) and voice hoarseness (%) were 37.2, 37.2, 37.2, 34.8 and 14, 14, 14,14 in group F at 1, 2, 6 and 24 hours, respectively, however, the p values were not found to be significant at any time interval. There was no significant difference in terms of severity of POST and voice hoarseness, patient satisfaction scores between the groups and there were no reported adverse events.

**Conclusions:** In patients undergoing nasosinus surgery under general anesthesia with endotracheal intubation, fluticasone furoate-impregnated throat packs failed to show any significant reduction in the incidence and severity of POST as well as hoarseness of voice, and even though it was not statistically significant, the fluticasone impregnated group had higher patient satisfaction scores.

**Registration:** CTRI ( CTRI/2020/09/027946; 22/09/2020).

## Introduction

Tracheal intubation is mostly performed under general anesthesia to secure the airway and is usually associated with variable degrees of trauma. Postoperative sore throat (POST), which ranges from 12.1% to 70%, is one of the commonest consequences of intubation, while hoarseness of voice varies from 4–43%. ^1^^–^^3^ The eighth worst possible post-anesthesia effect is POST, which imparts a strongly negative influence on the overall experience of surgery and stay in the hospital postoperatively. ^4^ POST is caused by a number of different factors, including vocal cord damage, congestive blood loss, and damage to the epithelium and mucosal cells caused by airway secretion. ^5^ In recent years, it has been reported that the shape of tracheal tubes, the size of the cuffs, the endotracheal intubation technique, the cuff pressure, and the use of inhalation anesthesia as contributing factors to POST. ^6^^–^^11^

Many surgical subspecialties use throat packs for various reasons. During surgery, they gather blood, secretions, and bone and cartilage fragments. It is asserted that doing so will lessen their inhalation and swallowing after surgery, which will lessen the likelihood of postoperative complications. ^12^ Even though there are several advantages of inserting throat packs there are certain drawbacks and POST is major among them. ^13^

To reduce the frequency of POST, many interventions can be used, including the use of compact tracheal tubes, ^14^ using video laryngoscopy during intubation, ^15^ limiting endotracheal cuff pressure, ^16^ using steroids during surgery, ^17^^–^^19^ topically applying non-steroidal anti-inflammatory drugs (NSAIDs), ^20^ or using different gargles (magnesium and ketamine) during surgery. ^21^

The topical administration of nonsteroidal anti-inflammatory drugs, lidocaine, steroids, N-methyl-d-aspartate receptor antagonists, and *Glycyrrhiza* have all been used to prevent POST through various mechanisms of action, according to systematic reviews. ^22^ However, a thorough search of the literature showed no evidence of studies involving the use of throat packs soaked with steroid sprays. ^23^ Our study drug, fluticasone furoate is typically available as a nasal spray and studies have not shown any adverse effects associated with the application of this spray. Therefore, the specific objectives of our study was to determine the effect of fluticasone-impregnated throat packs *versus* saline throat packs on the incidence and severity of POST and hoarseness of voice as well as to assess patient satisfaction at 24 hours in the postoperative period and any adverse events.

## Methods

### Ethical approval

This clinical trial received approval from the Institutional Ethics Committee (Kasturba Medical College and Kasturba Hospital Institutional Ethics Committee (IEC no: 714/2019, dated 18/09/2019) and was registered with the Clinical Trials Registry - India (CTRI; Registration no: CTRI/2020/09/027946; 22/09/2020). Patients provided written informed consent.

### Trial design

Between September 2020 and July 2021, patients scheduled for nasosinus surgeries under general anesthesia with endotracheal intubation at Kasturba Medical College and Hospital participated in this prospective, 1:1 randomized, double-blinded clinical trial. After receiving approval from the Kasturba Medical College and Kasturba Hospital Institutional Ethics Committee and registering with the CTRI, 86 patients were enrolled using the inclusion and exclusion criteria after providing written informed consent without any deviation from the original trial protocol and the trial was conducted according to the principles expressed in the Declaration of Helsinki. This study adheres to the CONSORT guidelines, and the proforma, model consent form and protocol can be found as *Extended data.* ^39^

### Participants

Participants classified as I or II according to the American Society of Anaesthesiologists (ASA) physical status classification, patients aged 18–60 years of both sexes were included in the study. Whereas those with a pre-existing sore throat and/or hoarseness of voice, anticipated difficult airway, already on steroids, requiring more than two attempts at endotracheal intubation and trauma during intubation were excluded.

Simple randomization with a 1:1 allocation ratio was carried out using an online randomization service. After patients had finished all baseline assessments and observer one had been blinded, sequentially numbered, opaque, and sealed envelopes were opened to reveal the allocation sequence.

### Interventions

Patient preparation: On the morning of operation, all patients were given tab ranitidine 150 mg and tab metoclopramide 10 mg. Nil per oral orders of 6 hours for solids and 2 hours for clear fluids was advised. The patient was identified on the day of surgery, fasting status was confirmed and they were shifted to the operating room. The monitors included a 5-electrode ECG, a non-invasive blood pressure monitor, and a pulse oximeter before induction of anaesthesia. Baseline blood pressure, heart rate and peripheral oxygen saturation were noted. The intravenous line was secured with a suitable gauge intravenous catheter under aseptic precautions, and the Ringer’s lactate infusion was started.

All the patients were pre-oxygenated for 3 minutes with 100% oxygen before being induced with intravenous 2 μg/kg fentanyl, induction with 2-3 mg/kg propofol, and neuromuscular blockade (NMB) with 0.1 mg/kg vecuronium. When the train-of-four count was zero, the patient was intubated under direct laryngoscopy (7 mm tube for females and 8 mm tube for males), which was inflated with air to a pressure of 25 mmHg. The demographic characteristics in terms of age, sex, BMI, smoking history and the mean duration of surgery were analyzed.

Observer one performed a preoperative evaluation and obtained informed consent. He was blinded to the group the patient was allotted. Additionally, he evaluated the patient after surgery and recorded the occurrence and severity of POST, voice hoarseness, and patient satisfaction score based on a predetermined score. Observer two performed the intubation and intervention according to the group allotted. Based on the group allocated, the patient received the throat pack soaked with either four puffs (400 mcg) of fluticasone furoate spray for group F and normal saline for group C. With the aid of Magill’s forceps and direct laryngoscopy, packs with standard dimensions of 120 cm × 7.5 cm were inserted. The use of steroids was avoided intraoperatively. Anesthesia was maintained with isoflurane, 40% oxygen and 60% air. Cuff pressure was kept below 25 cm H _2_O throughout the operation. In the end, the anesthetic agents were tapered and stopped, and 0.05 mg/kg of neostigmine was used to counter residual NMB. None of the patients received glycopyrrolate preoperatively or during the procedure. The throat pack was removed under direct laryngoscopy once the surgery was completed after gentle oral suctioning. Once the extubation criteria were satisfied, the patient was extubated and moved to the postoperative recovery room.

### Parameters assessed

• Age, sex, BMI, smoking history and the mean duration of surgery were documented.
• Post-operative sore throat at 1 hour, 2 hours, 6 hours and 24 hours using a 4-point scale.
• Hoarseness of voice at 1 hour, 6 hours and 24 hours using a 4-point scale.
• Patient satisfaction score at 24 hours in the postoperative period.

Observer 1 interviewed patients at 1, 2, 6, and 24 hours to assess the incidence and severity of postoperative sore throat and hoarseness of voice, patient satisfaction level as well as any adverse events.

### Postoperative assessment

The post-operative sore throat severity ^18^ was graded as follows: i) Grade 0, no sore throat at any time since the operation; ii) Grade 1, no pain, only discomfort or itchy sensation in the throat; iii) Grade 2, pain on swallowing or attempt at swallowing; and iv) Grade 3, pain at rest.

The severity of the hoarseness of voice ^19^ was graded as follows: i) Grade 0, no evidence of hoarseness of voice at any time since the operation; ii) Grade 1, no evidence of hoarseness at the time of the interview; iii) Grade 2, hoarseness at the time of interview noted by the patient only; and iv) Grade 3, hoarseness that is easily noted by the assessor at the time of the interview.

Patient satisfaction score was graded as follows: i) Score 0, poor; ii) Score 1, fair; iii) Score 2, good; and iv) Score 3, excellent.

### Outcomes

The primary outcome analyzed was the incidence of POST and post-operative voice hoarseness at 1, 2, 6 and 24 hours in both group C and group F. The secondary outcomes analyzed were severity of POST and voice hoarseness at 1, 2, 6 and 24 hours in both the groups including patient satisfaction scores at 24 hours post-surgery and any adverse events.

### Blinding

Observer one was the principal investigator who did the preoperative evaluation, after checking suitability for inclusion criteria in the study and obtaining written informed consent. He was blinded to the group the patient was allotted. He explained to the patient about the study and assessed the patient postoperatively on their grade of postoperative sore throat, hoarseness of voice and patient satisfaction score according to a pre-existing scoring scale.

Observer two was the Consultant anaesthesiologist, who performed the laryngoscopy and intubation and performed the intervention according to the group allotted.

### Statistical analysis

A pilot study with 10 patients in each group revealed that the control group had a 60% incidence of POST and the experimental group had a 40% incidence, which was used to determine the sample size. We required 96 patients in each group with a 20% difference between the two groups, assuming 85% power and a 5% level of significance. However, given the ongoing COVID-19 pandemic, due to a drastic reduction in the number of elective nasosinus surgeries in our institute, we were able to conduct our trial only in 86 patients (43 per group), despite extending our study period for three more months. For continuous variables, the data were presented as mean, and for categorical variables, as a percentage of frequency. To analyze significant differences between dichotomous variables, the chi-squared test was used. The term “p-value <0.05” denoted a statistically significant result. IBM SPSS Statistics (RRID:SCR_016479) 20.0 for Windows (SPSS Inc., Chicago, IL, US), a statistical software program, was used to conduct the statistical analysis.

## Results

**Figure 1.**
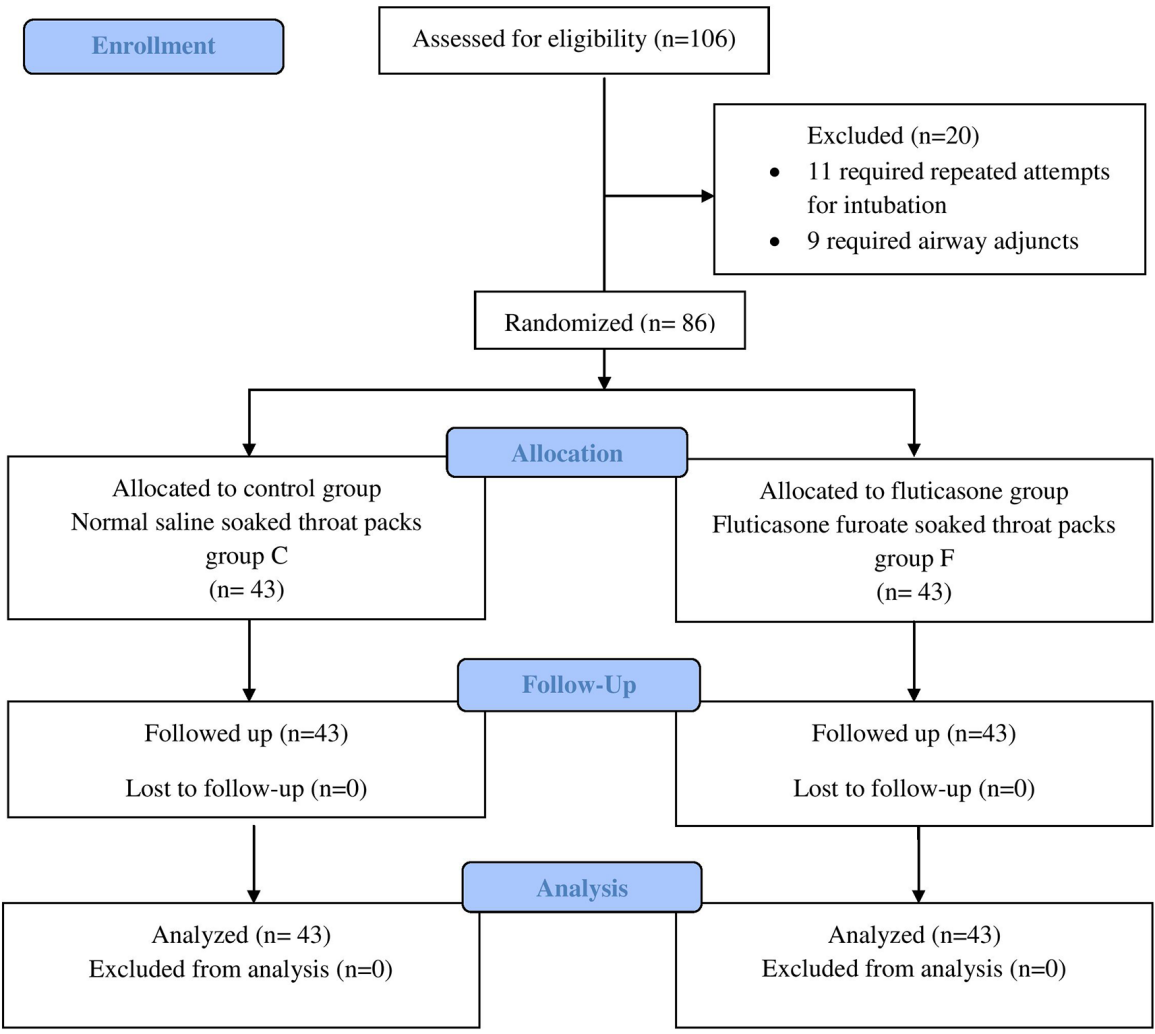
CONSORT diagram. Group C, Control group; Group F, Fluticasone group.

A total of 106 ASA I–II patients were screened in our study of which only 86 patients were included in our study. Of the 20 excluded, 11 patients required repeated attempts for intubation while nine required airway adjuncts ( Figure 1).

There were 43 patients in each group and their demographic characteristics were comparable with no significant difference in terms of age, sex, and smoking history. The majority of patients in either group had ASA physical status II. The mean duration of surgery among both groups was also similar ( Table 1).

**Table 1.** Baseline characteristics.

Characteristics	Group C (Mean ± SD) (n=43)	Group F (Mean ± SD) (n=43)	p-value
**Age (in years)**	29.2 ± 3.9	29.8 ± 3.5	0.52
**Female participants**	25	26	0.43
**BMI (kg/m** ^**2**^ **)**	27.4 ± 3.9	26.4 ± 2.9	0.3
**Smoking history**	13	15	0.63
**Duration of surgery (hours)**	2.3 ± 0.3	2.4. ± 0.3	0.92

Group C, control group; Group F, Fluticasone group; SD, Standard deviation; BMI, body mass index.

### Recruitment

Between September 2020 and July 2021, patients scheduled for nasosinus surgeries under general anesthesia with endotracheal intubation at Kasturba Medical College and Hospital participated in this prospective, 1:1 randomized, double-blinded clinical trial.

### Primary outcome

**Figure 2.**
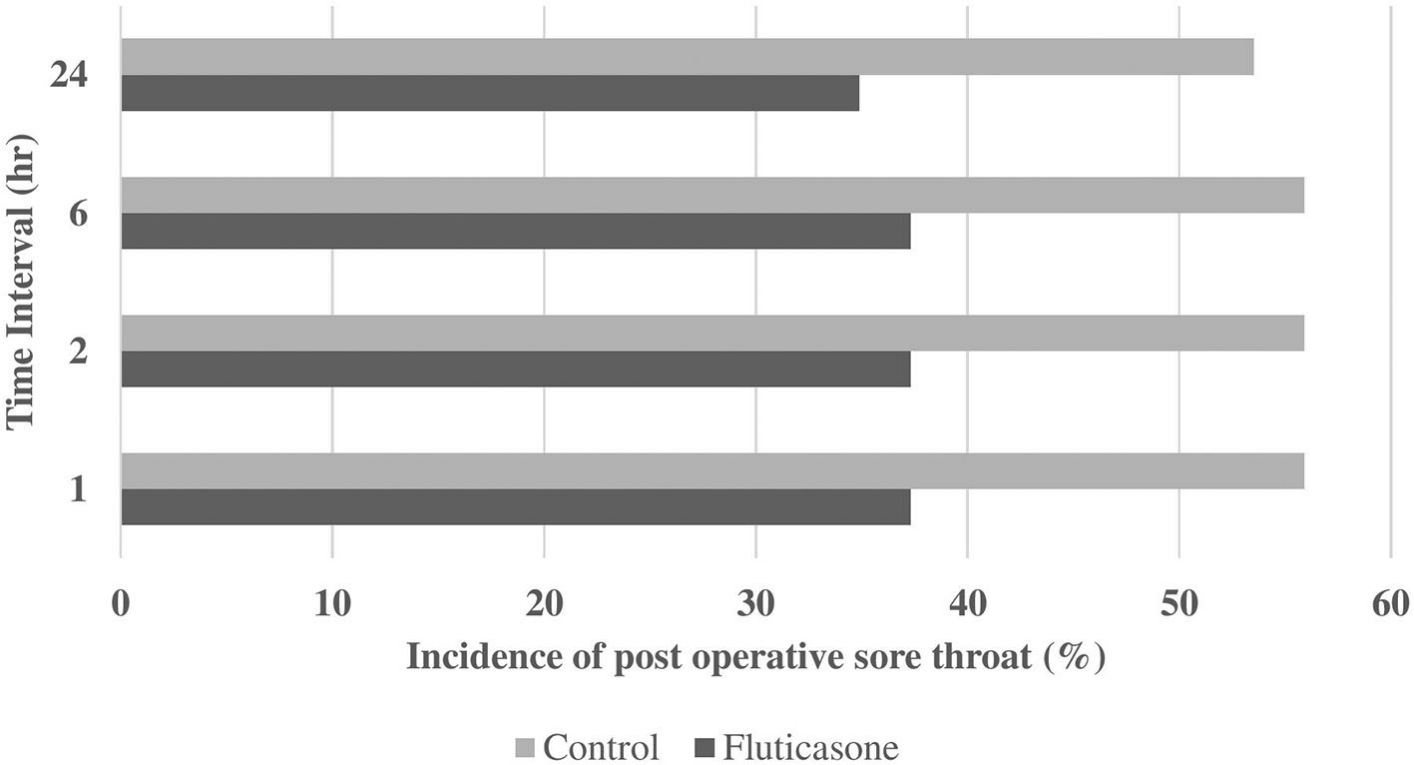
Incidence of POST. POST, postoperative sore throat.

**Figure 3.**
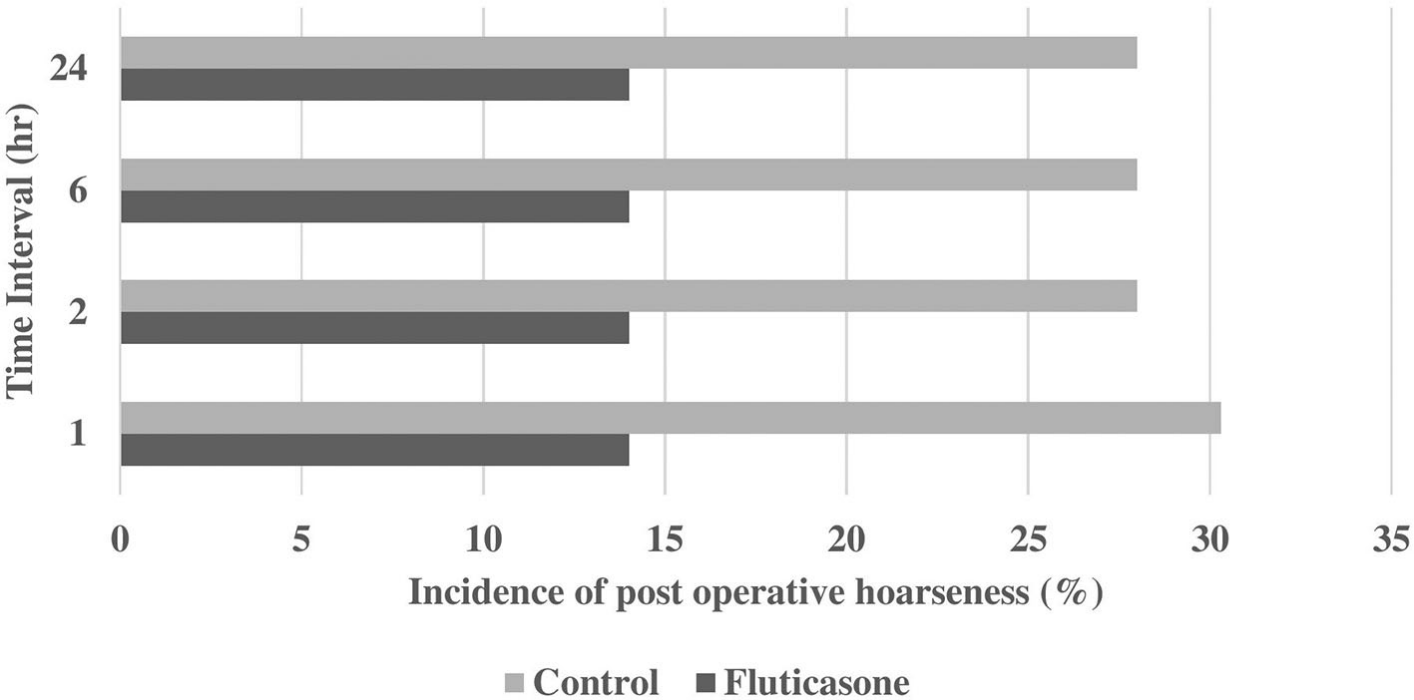
Incidence of postoperative hoarseness of voice.

Incidences of postoperative sore throat were 55.8, 55.6, 55.8 and 53.4% in group C at 1, 2, 6 and 24 hours and 37.2, 37.2, 37.2 and 34.8% in group F at 1, 2, 6 and 24 hours, respectively ( Table 2, Figure 2). Incidences of postoperative hoarseness of voice were found to be 30.2, 28.0, 28.0 and 28.0% in group C at 1, 2, 6 and 24 hours and 14.0, 14.0, 14.0 and 14.0% in the group F at 1, 2, 6 and 24 hours, respectively ( Table 2, Figure 3). Although there was a lower incidence of POST and hoarseness of voice in the fluticasone group at 1, 2, 6 and 24 hours, the p values were not found to be significant at any time interval.

**Table 2.** Primary outcomes.

Time interval (in hours)	Incidence of POST		p-value	Incidence of postoperative hoarseness of voice		p-value
	Group C (n=43) (n, % (95% CI))	Group F (n=43) (n, % (95% CI))		Group C (n=43) (n, % (95% CI))	Group F (n=43) (n, % (95% CI))	
1	24 (55.8%) (0.411, 0.695)	16 (37.2%) (0.243, 0.521)	0.52	13 (30.2%) (0.185, 0.452)	6 (14%) (0.061, 0.276)	0.11
2	24 (55.8%) (0.411, 0.695)	16 (37.2%) (0.243, 0.521)	0.52	12 (28%) (0.185, 0.452)	6 (14%) (0.061, 0.276)	0.18
6	24 (55.8%) (0.411, 0.695)	16 (37.2%) (0.243, 0.521)	0.52	12 (28%) (0.185, 0.452)	6 (14%) (0.061, 0.276)	0.18
24	23 (53.4%) (0.389, 0.674)	15 (34.8%) (0.223, 0.498)	0.12	12 (28%) (0.185, 0.452)	6 (14%) (0.061, 0.276)	0.18

POST, Post-operative sore throat; Group C, Control group; Group F, Fluticasone group; CI, Confidence interval.

### Secondary outcomes

According to the criteria outlined in the methodology, the degree of POST and hoarseness of voice were graded.

Grade 0 sore throats were present in 20 patients in group F (46.5%) and 28 patients in group C (65.1%). Grade 1 sore throats were present in 14 patients in group C (32.5%), 10 patients in group F (23.2%), and seven patients in group C (16.2%) and five patients in group F (11.6%). At 24 hours after surgery, only two patients in group C (4.6%) and none of the patients in group F had grade 3 sore throats. Nevertheless, there was no statistically significant difference between the groups ( Table 3).

**Table 3.** Secondary outcomes.

Grading of severity at 24 hours	Severity of POST		p-value	Severity of postoperative hoarseness of voice		p-value
	Group C (n=43) (n, % (95% CI))	Group F (n=43) (n, % (95% CI))		Group C (n=43) (n, % (95% CI))	Group F (n=43) (n, % (95% CI))	
0	28 (65.1%) (0.501, 0.776)	20 (46.5%) (0.325, 0.610)	0.072	23 (53.4%) (0.389, 0.674)	18 (41.8%) (0.283, 0.566)	0.099
1	14 (32.5%) (0.204, 0.475)	10 (23.2%) (0.129, 0.379)		10 (23.2%) (0.129, 0.379)	9 (20.9%) (0.112, 0.354)	
2	7 (16.2%) (0.078, 0.302)	5 (11.6%) (0.046, 0.249)		14 (32.5%) (0.204, 0.475)	10 (23.2%) (0.129, 0.379)	
3	2 (4.6%) (0.004, 0.163)	0		1 (2.3%) (0.0001, 0.131)	1 (2.3%) (0.0001, 0.131)	

POST, Post-operative sore throat; Group C, Control group; Group F, Fluticasone group; CI, Confidence interval.

A total of 23 patients in group C (53.4%) and 18 patients in group F (41.8%) both experienced grade 0 hoarseness of the voice. Grade 1 was present in 10 patients in group C (23.2%) and nine patients in group F (20.9%). Grade 2 hoarseness of voice affected 14 patients in group C (32.5%) and 10 patients in group F (23.2%). At 24 hours after surgery, there was one patient in group C (2.3%) and one patient in group F (2.3%) who both had grade 3 hoarseness of voice. The difference between the groups was present, but it was not statistically significant ( Table 3).

Most of the patients in both groups had excellent patient satisfaction scores, however, patients in group F had higher satisfaction scores 24 hours postoperatively but were not statistically significant ( Table 4).

**Table 4.** Patient satisfaction score at 24 hours.

Score	Group C (n = 43)	Group F (n = 43)	P-value
**0**	6	2	0.072
**1**	7	6	
**2**	9	6	
**3**	21	29	

Group C, Control group; Group F, Fluticasone group.

### Adverse events

Neither group experienced any adverse effects.

## Discussion

Endotracheal intubation is the most definitive way of securing the airway. Many anesthesiologists consider POST, a frequent anesthetic complication, to be a minor complication. However, for some patients, it might be their initial complaint as soon as they regain consciousness, hence reduction of POST enhances postoperative patient comfort, satisfaction and overall hospital stay. ^24^

Throat packs are used in oronasal procedures mainly to prevent intraoperative aspiration of blood into the respiratory and digestive tract. It is common knowledge that using pharyngeal packs makes recovery from surgery more painful and uncomfortable. POST brought on by oropharyngeal packs is unclear. It is postulated that the hard, abrasive cotton fibers of the pack can cause dryness of the surrounding tissues as well as the instrumentation per se can cause local inflammation eventually leading to POST and hoarseness of voice. ^25^ Even though various pharmacological modalities of attenuating POST have been studied in the past ^14^^–^^21^ there is no extensive literature documenting the use of steroid-impregnated throat packs. Since steroids have an anti-inflammatory effect, we analyzed their effect in mitigating POST and hoarseness of voice.

In prior studies, patient factors predicting POST included young age, being female and having a smoking history. ^26^^,^^27^ Lautenbacher *et al.*, ^28^ and Petrini *et al.*, ^29^ revealed that older patients when compared to the younger age group had a lesser response to pain stimulus as ageing reduces pain sensitivity and intensity, thus suggesting young age as a predictor of POST. However, in our study, we found no difference among the groups as the majority of the patients in both groups were young. According to Feine *et al.*, ^30^ women are more sensitive to the intensity of the pain and men typically have higher pain tolerance thresholds ^28^ suggesting that female patients tend to have a higher incidence of POST. Similar to the study by Jaensson *et al.*, ^31^ who found no appreciable difference in POST incidence between men and women, there is no effect of sex on POST and hoarseness of voice in this study. Smoking is a significant predictor because it is known to cause airway inflammation on its own, ^32^^,^^33^ which contributes to POST, however, due to the small sample size in this study, we were unable to detect any influence on POST, which is similar to the study by Lee *et al.* ^34^

In the study by Higgins *et al.*, ^2^ he found that the incidence of POST was higher in those with ASA physical status III when compared to those with ASA I/II (p≤0.05), however in our study all the patients had ASA status of I/II, thus no difference could be recorded. Longer surgery times have been shown in earlier studies ^35^^,^^36^ to affect the occurrence of POST, with factors including surgical manipulation of the airway and surrounding tissue, repeated suctioning, high anesthetic gas flow rates, and lack of airway humidity as contributors. However, in our study, there was no such difference because all of the patients were posted for nasosinus surgery and the surgical times in the two groups were comparable.

The overall incidence of POST and hoarseness of voice in our study was similar to the study done in 2017 on Korean patients by Lee *et al.*, ^34^ wherein 207 patients were analyzed, among whom 119 patients (57.5%) developed POST and 80 patients (38.6%) developed hoarseness of voice. However, many prior studies have shown that the incidence of POST may be up to 70% and that of hoarseness is reported to range from 4–43%. ^1^^–^^4^ Even though the incidence of POST and hoarseness of voice was lower in the fluticasone group than in the control group in this study, there was never a postoperative difference that was statistically significant. This study is comparable to one by Park SY *et al.*, ^37^ which compared the incidence of POST in patients with tracheal tubes impregnated with triamcinolone acetonide (n=72) and chlorhexidine (n=72) during intubation. His research demonstrated that when compared to the chlorhexidine group (71.8%; 51/72) the incidence of POST was significantly lower in the steroid-impregnated group (19.4%; 14/72) (p<0.001).

The overall severity of POST as well as hoarseness of voice was lower fluticasone group but was not statistically significant. These results are similar to the study by Park SY *et al.*, ^37^ wherein the severity of POST was analyzed at 1, 6 and 24 hours and found that among 72 patients in the steroid-soaked throat pack group majority (68/72) had grade 0 severity at 1 hour and 24 hours and very few (10/72) had grade 1 severity of POST, which is similar to our study, however, their study revealed significant difference (p<0.001), which is unlike our study and probably can be explained by the small sample size in each group.

The majority of patients in this study reported positive experiences with fluticasone-impregnated throat packs when compared to the placebo group, but the findings were not statistically significant. The analysis of POST in 140 patients who needed intubation by Macintosh laryngoscope *versus* GlideScope in the study by Aqil M *et al.*, ^38^ also included patient satisfaction scores at 24 hours following surgery, which was similar to our study.

### Limitations

This was a single center study with a relatively small sample size. Our study included only nasosinus surgeries and with respect to the endotracheal tube, the number of attempts, time taken for insertion and cuff pressure were not analyzed in this study.

### Generalisability

Randomized controlled trials (RCTs) provide the highest level of evidence. In this study we utilised the anti-inflammatory property of fluticasone furoate on reducing POST, which is one of the most disturbing after effects post-endotracheal intubation. Even though our study showed lesser incidence and severity of POST as well as hoarseness of voice it was not statistically significant, which might be due to small sample size in each group. Thus, after proving with an adequate sample size, our intervention could be applicable and would be beneficial in reducing the incidence of POST in patients undergoing endotracheal intubation.

## Conclusions

The frequency and severity of POST and hoarseness of voice in patients undergoing nasosinus surgery under general anesthesia with endotracheal intubation were not significantly reduced by fluticasone furoate-impregnated throat packs. It gave a higher satisfaction score after 24 hours even though it was statistically insignificant.

## Data Availability

Due to the fact that open posting of data on a repository was not included in the study information sheet at the time the study was done, data access will be granted once users have consented to the data sharing agreement and provided written plans and justification for what is proposed with the data. Data access may be obtained by submitting a request to the corresponding author. Figshare: Protocol, https://doi.org/10.6084/m9.figshare.23850630.v4. ^39^ This project contains the following extended data:
- CONSORT Checklist.doc - Proforma.docx - Informed consent.docx - Protocol.doc Data are available under the terms of the Creative Commons Attribution 4.0 International license (CC-BY 4.0).

## References

[1] Tanaka Y Nakayama T Nishimori M : Lidocaine for preventing postoperative sore throat. *Cochrane Database Syst. Rev.* 2015;7. 10.1002/14651858.CD004081.pub3 PMC7151755 26171894

[2] Higgins PP Chung F Mezei G : Postoperative sore throat after ambulatory surgery. *Br. J. Anaesth.* 2002 Apr 1;88(4):582–584. 10.1093/bja/88.4.582 12066737

[3] Sharma S Bhardwaj V Sharma S : Dexamethasone to decrease post-anesthesia sore throat (POST) and hoarseness-which is the most effective route: intravenous, topical, or nebulization? A prospective randomized trial. *Ain-Shams J. Anesthes.* 2021 Dec;13(1):1–7. 10.1186/s42077-021-00144-8

[4] El-Boghdadly K Bailey CR Wiles MD : Postoperative sore throat: a systematic review. *Anaesthesia.* 2016 Jun;71(6):706–717. 10.1111/anae.13438 27158989

[5] Winkel E Knudsen J : Effect on the incidence of postoperative sore throat of 1 percent cinchocaine jelly for endotracheal intubation. *Anesth. Analg.* 1971 Jan 1;50(1):92–94. 10.1213/00000539-197101000-00018 5100250

[6] Saeki H Morimoto Y Yamashita A : Postoperative sore throat and intracuff pressure: comparison among endotracheal intubation, laryngeal mask airway and cuffed oropharyngeal airway. *Masui.* 1999 Dec 1;48(12):1328–1331. 10658413

[7] Lee J Park HP Jeong MH : Combined intraoperative paracetamol and preoperative dexamethasone reduces postoperative sore throat: a prospective randomized study. *J. Anesth.* 2017 Dec;31:869–877. 10.1007/s00540-017-2411-6 28980140

[8] Hu B Bao R Wang X : The size of endotracheal tube and sore throat after surgery: a systematic review and meta-analysis. *PLoS One.* 2013 Oct 4;8(10):e74467. 10.1371/journal.pone.0074467 24124452 PMC3790787

[9] Chang JE Kim H Han SH : Effect of endotracheal tube cuff shape on postoperative sore throat after endotracheal intubation. *Anesth. Analg.* 2017 Oct 1;125(4):1240–1245. 10.1213/ANE.0000000000001933 28368938

[10] Sato K Tanaka M Nishikawa T : Changes in intracuff pressure of endotracheal tubes permeable or resistant to nitrous oxide and incidence of postoperative sore throat. *Masui.* 2004 Jul 1;53(7):767–771. 15298243

[11] Joe HB Kim DH Chae YJ : The effect of cuff pressure on postoperative sore throat after Cobra perilaryngeal airway. *J. Anesth.* 2012 Apr;26:225–229. 10.1007/s00540-011-1293-2 22127511 PMC3328671

[12] Fennessy BG Mannion S Kinsella JB : The benefits of hypopharyngeal packing in nasal surgery: a pilot study. *Ir. J. Med. Sci.* 2011 Mar;180:181–183. 10.1007/s11845-010-0601-4 21110138

[13] Karbasforushan A Hemmatpoor B Makhsosi BR : The effect of pharyngeal packing during nasal surgery on the incidence of post-operative nausea, vomiting, and sore throat. *Iran J. Otorhinolaryngol.* 2014 Oct;26(77):219–223. 25320699 PMC4196445

[14] Zhang W Zhao G Li L : Prophylactic administration of corticosteroids for preventing postoperative complications related to tracheal intubation: a systematic review and meta-analysis of 18 randomized controlled trials. *Clin. Drug Investig.* 2016 Apr;36:255–265. 10.1007/s40261-015-0369-4 26715108

[15] Najafi A Imani F Makarem J : Postoperative sore throat after laryngoscopy with macintosh or glide scope video laryngoscope blade in normal airway patients. *Anesthesiol. Pain Med.* 2014 Feb;3(1). 10.5812/aapm.15136 PMC3961026 24660157

[16] Ansari L Bohluli B Mahaseni H : The effect of endotracheal tube cuff pressure control on postextubation throat pain in orthognathic surgeries: a randomized double-blind controlled clinical trial. *Br. J. Oral Maxillofac. Surg.* 2014 Feb 1;52(2):140–143. 10.1016/j.bjoms.2013.10.005 24268872

[17] Sun L Guo R Sun L : Dexamethasone for preventing postoperative sore throat: a meta-analysis of randomized controlled trials. *Ir. J. Med. Sci.* 2014 Dec;183:593–600. 10.1007/s11845-013-1057-0 24357270

[18] Canbay O Celebi N Sahin A : Ketamine gargle for attenuating postoperative sore throat. *Br. J. Anaesth.* 2008 Apr 1;100(4):490–493. 10.1093/bja/aen023 18310675

[19] Kadar MA : Assessment of the efficacy of dexamethasone, lignocaine or placebo in the prevention of post intubation sore throat. *Int. J. Biomed. Res.* 2015;6(07):493–503. 10.7439/ijbr.v6i7.2310

[20] Chang JE Min SW Kim CS : The effect of benzydamine hydrochloride prophylaxis on postoperative sore throat and hoarseness after tracheal intubation using a double-lumen endobronchial tube: a randomized controlled trial. *Can. J. Anaesth.* 2015 Oct;62:1097–1103. 10.1007/s12630-015-0432-x 26149601

[21] Teymourian H Mohajerani SA Farahbod A : Magnesium and ketamine gargle and postoperative sore throat. *Anesth. Pain Med.* 2015 Jun;5(3):e22367. 10.5812/aapm.5(3)2015.22367 26161316 PMC4493740

[22] Wang G Qi Y Wu L : Comparative efficacy of 6 topical pharmacological agents for preventive interventions of postoperative sore throat after tracheal intubation: A systematic review and network meta-analysis. *Anesth. Analg.* 2021 Jul;133(1):58–67. 10.1213/ANE.0000000000005521 33886521 PMC8183478

[23] Thompson MJ Hayward G Heneghan CJ : Corticosteroids for sore throat. *Cochrane Database Syst. Rev.* 2010;1. 10.1002/14651858.CD008268 23076943

[24] Smarius BJ Guillaume CH Jonker G : The use of throat packs in pediatric cleft lip/palate surgery: a retrospective study. *Clin. Oral Investig.* 2018 Dec;22:3053–3059. 10.1007/s00784-018-2387-0 29473105 PMC6224011

[25] Bajwa SJ : Prevention of aspiration of blood with a unique pharyngeal packing method. *Anesth. Essays Res.* 2012 Jul;6(2):251–252. 10.4103/0259-1162.108361 25885633 PMC4173445

[26] Mencke T Echternach M Kleinschmidt S : Laryngeal morbidity and quality of tracheal intubation: a randomized controlled trial. *The Journal of the American Society of Anesthesiologists.* 2003 May 1;98(5):1049–1056. 10.1097/00000542-200305000-00005 12717124

[27] Biro P Seifert B Pasch T : Complaints of sore throat after tracheal intubation: a prospective evaluation. *Eur. J. Anaesthesiol.* 2005 Apr;22(4):307–311. 10.1017/S0265021505000529 15892411

[28] Lautenbacher S Peters JH Heesen M : Age changes in pain perception: a systematic-review and meta-analysis of age effects on pain and tolerance thresholds. *Neurosci. Biobehav. Rev.* 2017 Apr 1;75:104–113. 10.1016/j.neubiorev.2017.01.039 28159611

[29] Petrini L Matthiesen ST Arendt-Nielsen L : The effect of age and gender on pressure pain thresholds and suprathreshold stimuli. *Perception.* 2015 May;44(5):587–596. 10.1068/p7847 26422905

[30] Feine JS Bushnell MC Miron D : Sex differences in the perception of noxious heat stimuli. *Pain.* 1991 Mar 1;44(3):255–262. 10.1016/0304-3959(91)90094-E 2052394

[31] Jaensson M Gupta A Nilsson U : Gender differences in sore throat and hoarseness following endotracheal tube or laryngeal mask airway: a prospective study. *BMC Anesthesiol.* 2014 Dec;14(1):1–8. 10.1186/1471-2253-14-56 25061426 PMC4110067

[32] Jayes L Haslam PL Gratziou CG : SmokeHaz: systematic reviews and meta-analyses of the effects of smoking on respiratory health. *Chest.* 2016 Jul 1;150(1):164–179. 10.1016/j.chest.2016.03.060 27102185

[33] Thomson NC : Asthma and smoking-induced airway disease without spirometric COPD. *Eur. Respir. J.* 2017 May 1;49(5):1602061. 10.1183/13993003.02061-2016 28461294

[34] Lee JY Sim WS Kim ES : Incidence and risk factors of postoperative sore throat after endotracheal intubation in Korean patients. *J. Int. Med. Res.* 2017 Apr;45(2):744–752. 10.1177/0300060516687227 28173712 PMC5536682

[35] Patil BO Sonavdekar SR Mathur R : A Relative Study on Laryngeal Mask Airway Lubrication with 0.005% Beclomethasone Cream v/a 2% Lidocaine.

[36] Sultan SS Fahmy NM Hassan MI : Soaking oro-pharyngeal pack with triamcinolone acetonide lowers discomfort in functional endoscopic sinus surgeries. *Rev. Chil. Anest.* 2020;49:889–895. 10.25237/revchilanestv49n06-15

[37] Park SY Lee SJ : Application of triamcinolone acetonide paste to the endotracheal tube reduces postoperative sore throat: a randomized controlled trial. *Can. J. Anesth.* 2011 May 1;58(5):436–442. 10.1007/s12630-011-9478-6 21359615

[38] Aqil M Khan MU Mansoor S : Incidence and severity of postoperative sore throat: a randomized comparison of Glidescope with Macintosh laryngoscope. *BMC Anesthesiol.* 2017 Dec;17(1):1–8. 10.1186/s12871-017-0421-4 28899338 PMC5596501

[39] Kanakalakshmi ST : Protocol.[Dataset]. *figshare.* 2023. 10.6084/m9.figshare.23850630.v4

