# Supplementary material for: Effect of fluticasone-impregnated throat packs on postoperative sore throat (POST) and hoarseness of voice: A randomized clinical trial
Source: F1000Res. 2024 Jun 10;12:1352. Originally published 2023 Oct 17. [Version 2] doi: 10.12688/f1000research.139742.2 (PMC10905143; doi:10.12688/f1000research.139742.2)
Supplement: Peer review history

# Reviewer report: Reviewer response for version 2

**DOI:** 10.5256/f1000research.166346.r325007

**Published:** 2024-10-25

**Authors:** Abdul Rahman Muthanna

**Affiliations:** 1 Universiti Putra Malaysia, Serdang, Selangor, Malaysia

This manuscript is well-written in term of method and focuses primarily on statistical analysis. However, there is minor correction, please revise accordingly.

 Abstract

 Background: "through various interventions" include example of the interventions or be specific.

 Methods: A total of respondents need to be included to each groups.

 Include study duration in methods.

 Include statistical analysis.

 Results: the results lack numerical values. It should have both words

 Conclusion: what is the recommendations.

 Introduction

 What is the limitations and recommendations from previous studies regarding the interventions. (Need to discuss).

 Methods 

 Include sample size calculation.

 Results

 Ok

 Discussion 

 Ok

Is the work clearly and accurately presented and does it cite the current literature?

Yes

If applicable, is the statistical analysis and its interpretation appropriate?

Yes

Are all the source data underlying the results available to ensure full reproducibility?

Yes

Is the study design appropriate and is the work technically sound?

Yes

Are the conclusions drawn adequately supported by the results?

Yes

Are sufficient details of methods and analysis provided to allow replication by others?

Yes

Reviewer Expertise:

Medical Microbiology

I confirm that I have read this submission and believe that I have an appropriate level of expertise to confirm that it is of an acceptable scientific standard.

# Reviewer report: Reviewer response for version 1

**DOI:** 10.5256/f1000research.153042.r237023

**Published:** 2024-02-09

**Authors:** Riniki Sarma

**Affiliations:** 1 All India Institute of Medical Sciences, Delhi, New Delhi, India

This randomized controlled trial takes into account an innovative drug to find the outcome of sore throat and hoarseness of voice postoperatively in  patients undergoing endotracheal intubation. Fluticasone is a known treatment method used in rhinosinusitis due to its anti-inflammatory properties. Though not statistically significant, in view of the incidence of POST being lesser with fluticasone group, the findings from this study are encouraging.

Is the work clearly and accurately presented and does it cite the current literature?

Yes

If applicable, is the statistical analysis and its interpretation appropriate?

Yes

Are all the source data underlying the results available to ensure full reproducibility?

Yes

Is the study design appropriate and is the work technically sound?

Yes

Are the conclusions drawn adequately supported by the results?

Yes

Are sufficient details of methods and analysis provided to allow replication by others?

Yes

Reviewer Expertise:

AIrway, resuscitation, Pain management

I confirm that I have read this submission and believe that I have an appropriate level of expertise to confirm that it is of an acceptable scientific standard.

# Response

**Published:** 2024-03-24

**Authors:** Sushma Thimmaiah Kanakalakshmi

**Affiliations:** Anesthesiology, Kasturba Hospital Manipal, Manipal, Karnataka, India

**Riniki Sarma**

 This randomized controlled trial takes into account an innovative drug to find the outcome of sore throat and hoarseness of voice postoperatively in patients undergoing endotracheal intubation. Fluticasone is a known treatment method used in rhinosinusitis due to its anti-inflammatory properties. Though not statistically significant, in view of the incidence of POST being lesser with fluticasone group, the findings from this study are encouraging.

**Response**

 Many thanks for your comments madam.

 Thank you for your motivating comments.

# Reviewer report: Reviewer response for version 1

**DOI:** 10.5256/f1000research.153042.r217314

**Published:** 2024-02-09

**Authors:** Mathew George

**Affiliations:** 1 Amrita Institute of Medical Sciences and Research Centre, Kochi, India

The study is well designed and executed. The background research is also commendable. If possible it would be better to continue patient enrolment to achieve the calculated sample size of 96 patients per group as it would possibly improve the power of the study and likely bring out a significant difference between the groups as there is a trend towards fluticasone decreasing the incidence of the primary outcome.

 The paragraph on secondary outcome needs some correction as the number of patients with grade 2 soreness is not mentioned appropriately, or has been missed.

 Altogether the paper is well designed and reasonably well written but is underpowered to reveal the true benefits if any for the treatment being studied.

Is the work clearly and accurately presented and does it cite the current literature?

Partly

If applicable, is the statistical analysis and its interpretation appropriate?

Yes

Are all the source data underlying the results available to ensure full reproducibility?

Yes

Is the study design appropriate and is the work technically sound?

Yes

Are the conclusions drawn adequately supported by the results?

Yes

Are sufficient details of methods and analysis provided to allow replication by others?

Yes

Reviewer Expertise:

Neuro anaesthesia, Paediatric Anaesthesia, Intraoperative evoked potential monitoring

I confirm that I have read this submission and believe that I have an appropriate level of expertise to confirm that it is of an acceptable scientific standard, however I have significant reservations, as outlined above.

# Response

**Published:** 2024-03-24

**Authors:** Sushma Thimmaiah Kanakalakshmi

**Affiliations:** Anesthesiology, Kasturba Hospital Manipal, Manipal, Karnataka, India

**Mathew George**

**Comment**

 The study is well designed and executed. The background research is also commendable. If possible it would be better to continue patient enrolment to achieve the calculated sample size of 96 patients per group as it would possibly improve the power of the study and likely bring out a significant difference between the groups as there is a trend towards fluticasone decreasing the incidence of the primary outcome.

**Response**

 Many thanks for your comments. We are continuing the patient enrollment as suggested so as to improve the power of our study and trying to find any significant differences between the groups.

**Comment**

 The paragraph on secondary outcome needs some correction as the number of patients with grade 2 soreness is not mentioned appropriately, or has been missed.

**Response**

 Many thanks for your comments. We have made the necessary changes and highlighted the same.

**Comment**

 Altogether the paper is well designed and reasonably well written but is underpowered to reveal the true benefits if any for the treatment being studied.

**Response**

 Many thanks for your comments. We agree with your suggestion and thus are continuing recruiting patients so as to improve the overall number and power of the study.

# Reviewer report: Reviewer response for version 1

**DOI:** 10.5256/f1000research.153042.r241363

**Published:** 2024-02-07

**Authors:** Ajinkya Pawar

**Affiliations:** 1 Department of Conservative Dentistry and Endodontics, Nair Hospital Dental College, Mumbai, Maharashtra, India

The introduction of the manuscript titled "Effect of fluticasone-impregnated throat packs on postoperative sore throat (POST) and hoarseness of voice: A randomized clinical trial" provides a comprehensive overview of the background, rationale, and objectives of the study. Overall, the introduction is well-structured and informative. However, here are some comments on potential flaws or areas for improvement:

 The introduction mentions the incidence of POST ranging from 12.1% to 70% and hoarseness from 4–43%. However, it would be helpful to provide the sources of these statistics to lend credibility to the claims. This is important for readers to evaluate the reliability and generalizability of the reported incidence rates.

 The statement that POST is the eighth worst possible post-anesthesia effect is intriguing, but the source or reference for this specific ranking is not provided.

 While the introduction mentions that throat packs have advantages, it briefly touches on the drawbacks, with POST being a major concern. Expanding on the drawbacks and potential complications associated with throat packs could provide a more balanced view and help readers understand the context for investigating alternative interventions.

 The introduction discusses various interventions to reduce the frequency of POST, such as compact tracheal tubes, video laryngoscopy, cuff pressure control, steroids, NSAIDs, and different gargles. It might be beneficial to briefly mention the effectiveness or limitations of these interventions.

 The introduction mentions a thorough search of the literature showing no evidence of studies involving the use of throat packs soaked with steroid sprays. While this highlights the novelty of the study, it would be helpful to elaborate on why this gap exists and why it is essential to address it.

 The introduction mentions that fluticasone furoate is typically available as a nasal spray with no reported adverse effects. While this information is relevant, it might be helpful to briefly explain why fluticasone furoate is being considered for impregnating throat packs, especially in comparison to other available steroids or anti-inflammatory agents.

 The specific objectives of the study are outlined at the end of the introduction, but the wording could be refined for clarity. Consider rephrasing to clearly state each objective and its significance in the context of the study.

**Discussion**

 The section provides a thorough analysis of the study results and offers valuable insights. However, there are a few areas that could be addressed for clarity and completeness:

 While the discussion emphasizes the significance of reducing POST for postoperative patient comfort, satisfaction, and overall hospital stay, it would be beneficial to delve deeper into the specific impact of POST on patients' experiences. Providing anecdotes or examples could help readers better understand the practical implications of mitigating POST.

 The discussion briefly mentions the use of steroids for their anti-inflammatory effect in preventing POST. It would be helpful to expand on the rationale behind choosing fluticasone furoate specifically, considering its anti-inflammatory properties and its potential advantages over other steroids. This could strengthen the justification for the study intervention.

 The discussion compares the results of the current study with findings from previous research, particularly the study by Park SY et al (Ref1). However, it would be beneficial to discuss any differences in study design, patient populations, or interventions that may contribute to variations in results.

 The limitations section provides essential information about the constraints of the study, such as the single-center design and relatively small sample size. However, there is a mention of not analyzing certain aspects related to endotracheal tubes, such as the number of attempts, time for insertion, and cuff pressure.

 The discussion touches upon the small sample size as a potential reason for the lack of statistical significance in certain outcomes. It would be beneficial to discuss the implications of the study's limitations on the generalizability of the findings. Additionally, addressing the types of patients or surgeries to which the results may be more applicable could enhance the discussion.

 Considering the study's findings and limitations, it would be helpful to suggest potential avenues for future research. This could include recommendations for larger multi-center trials, exploring different patient populations, or investigating variations in the intervention protocol.

 Ensure consistency in reporting findings. For instance, the discussion mentions a lower incidence and severity of POST in the fluticasone group, but then notes that the results were not statistically significant. Clarify whether these observed differences are clinically meaningful even if not statistically significant.

 Acknowledge the strengths of the study, such as the randomized controlled trial design and the use of a specific intervention that has not been extensively studied before. Recognizing these strengths reinforces the scientific rigor of the research.

Is the work clearly and accurately presented and does it cite the current literature?

Yes

If applicable, is the statistical analysis and its interpretation appropriate?

Yes

Are all the source data underlying the results available to ensure full reproducibility?

Yes

Is the study design appropriate and is the work technically sound?

Yes

Are the conclusions drawn adequately supported by the results?

Yes

Are sufficient details of methods and analysis provided to allow replication by others?

Yes

Reviewer Expertise:

Clinical Trial

I confirm that I have read this submission and believe that I have an appropriate level of expertise to confirm that it is of an acceptable scientific standard, however I have significant reservations, as outlined above.

## References

[1] : Application of triamcinolone acetonide paste to the endotracheal tube reduces postoperative sore throat: a randomized controlled trial. *Can J Anaesth* .2011;58(5) : 10.1007/s12630-011-9478-6 436-42 10.1007/s12630-011-9478-6 21359615

# Response

**Published:** 2024-05-10

**Authors:** Sushma Thimmaiah Kanakalakshmi

**Affiliations:** Anesthesiology, Kasturba Hospital Manipal, Manipal, Karnataka, India

The introduction of the manuscript titled "Effect of fluticasone-impregnated throat packs on postoperative sore throat (POST) and hoarseness of voice: A randomized clinical trial" provides a comprehensive overview of the background, rationale, and objectives of the study. Overall, the introduction is well-structured and informative.

 However, here are some comments on potential flaws or areas for improvement:

**Comment**

 The introduction mentions the incidence of POST ranging from 12.1% to 70% and hoarseness from 4–43%. However, it would be helpful to provide the sources of these statistics to lend credibility to the claims. This is important for readers to evaluate the reliability and generalizability of the reported incidence rates.

**Response**

 Many thanks for your suggestion. We have changed the prevalence of POST and hoarseness of voice and have mentioned them in the revised manuscript.

**Comment**

 The statement that POST is the eighth worst possible post-anesthesia effect is intriguing, but the source or reference for this specific ranking is not provided.

**Response**

 Many thanks for your suggestion. We have changed the statement and have mentioned in the revised manuscript.

**Comment**

 While the introduction mentions that throat packs have advantages, it briefly touches on the drawbacks, with POST being a major concern. Expanding on the drawbacks and potential complications associated with throat packs could provide a more balanced view and help readers understand the context for investigating alternative interventions.

**Response**

 Many thanks for your suggestion. We have added supporting study and have mentioned them in the revised manuscript.

**Comment**

 The introduction discusses various interventions to reduce the frequency of POST, such as compact tracheal tubes, video laryngoscopy, cuff pressure control, steroids, NSAIDs, and different gargles. It might be beneficial to briefly mention the effectiveness or limitations of these interventions.

**Response**

 Many thanks for your suggestion. We have added supporting studies and have mentioned in the revised manuscript.

**Comment**

 The introduction mentions a thorough search of the literature showing no evidence of studies involving the use of throat packs soaked with steroid sprays. While this highlights the novelty of the study, it would be helpful to elaborate on why this gap exists and why it is essential to address it.

**Response**

 Many thanks for your suggestion. We have added the supporting study and have mentioned in the revised manuscript.

**Comment**

 The introduction mentions that fluticasone furoate is typically available as a nasal spray with no reported adverse effects. While this information is relevant, it might be helpful to briefly explain why fluticasone furoate is being considered for impregnating throat packs, especially compared to other available steroids or anti-inflammatory agents.

**Response**

 Many thanks for your suggestion. We have added the supporting study and have mentioned it in the revised manuscript.

**Comment**

 The specific objectives of the study are outlined at the end of the introduction, but the wording could be refined for clarity. Consider rephrasing to clearly state each objective and its significance in the context of the study.

**Response**

 Many thanks for your suggestion. We have made the necessary changes and have mentioned them in the revised manuscript.

**Discussion**

 The section provides a thorough analysis of the study results and offers valuable insights.

 However, there are a few areas that could be addressed for clarity and completeness:

**Comment**

 While the discussion emphasizes the significance of reducing POST for postoperative patient comfort, satisfaction, and overall hospital stay, it would be beneficial to delve deeper into the specific impact of POST on patients' experiences. Providing anecdotes or examples could help readers better understand the practical implications of mitigating POST.

**Response**

 Many thanks for your suggestion. We have added the studies and have mentioned them in the revised manuscript.

**Comment**

 The discussion briefly mentions the use of steroids for their anti-inflammatory effect in preventing POST. It would be helpful to expand on the rationale behind choosing fluticasone furoate specifically, considering its anti-inflammatory properties and its potential advantages over other steroids. This could strengthen the justification for the study intervention.

**Response**

 Many thanks for your suggestion. We have made the necessary changes and have mentioned them in the revised manuscript.

**Comment**

 The discussion compares the results of the current study with findings from previous research, particularly the study by Park SY et al (Ref1). However, it would be beneficial to discuss any differences in study design, patient populations, or interventions that may contribute to variations in results.

**Response**

 Many thanks for your suggestion. We have made the necessary changes and have mentioned them in the revised manuscript.

**Comment**

 The limitations section provides essential information about the constraints of the study, such as the single-center design and relatively small sample size. However, there is a mention of not analyzing certain aspects related to endotracheal tubes, such as the number of attempts, time for insertion, and cuff pressure. The discussion touches upon the small sample size as a potential reason for the lack of statistical significance in certain outcomes. It would be beneficial to discuss the implications of the study's limitations on the generalizability of the findings. Additionally, addressing the types of patients or surgeries to which the results may be more applicable could enhance the discussion.

**Response**

 Many thanks for your suggestion. We have made the necessary changes and have mentioned them in the revised manuscript.

**Comment**

 Considering the study's findings and limitations, it would be helpful to suggest potential avenues for future research. This could include recommendations for larger multi-centre trials, exploring different patient populations, or investigating variations in the intervention protocol.

**Response**

 Many thanks for your suggestion. We have made the necessary changes and have mentioned them in the revised manuscript

**Comment**

 Ensure consistency in reporting findings. For instance, the discussion mentions a lower incidence and severity of POST in the fluticasone group, but then notes that the results were not statistically significant. Clarify whether these observed differences are clinically meaningful even if not statistically significant.

**Response**

 Many thanks for your suggestion. Although the observed differences were clinically meaningful they were not statistically significant as mentioned in our study.

**Comment**

 Acknowledge the strengths of the study, such as the randomized controlled trial design and the use of a specific intervention that has not been extensively studied before. Recognizing these strengths reinforces the scientific rigor of the research.

**Response**

 Many thanks for your suggestion. We have made the necessary changes and have mentioned them in the revised manuscript
